# Supplementary material for: Piezoelectric Gas Sensors with Polycomposite Coatings in Biomedical Application
Source: Sensors (Basel). 2022 Nov 5;22(21):8529. doi: 10.3390/s22218529 (PMC9654775; doi:10.3390/s22218529)
Supplement: Supplementary file 1 [file sensors-22-08529-s001.zip › sensors-1976696-supplementary.pdf]

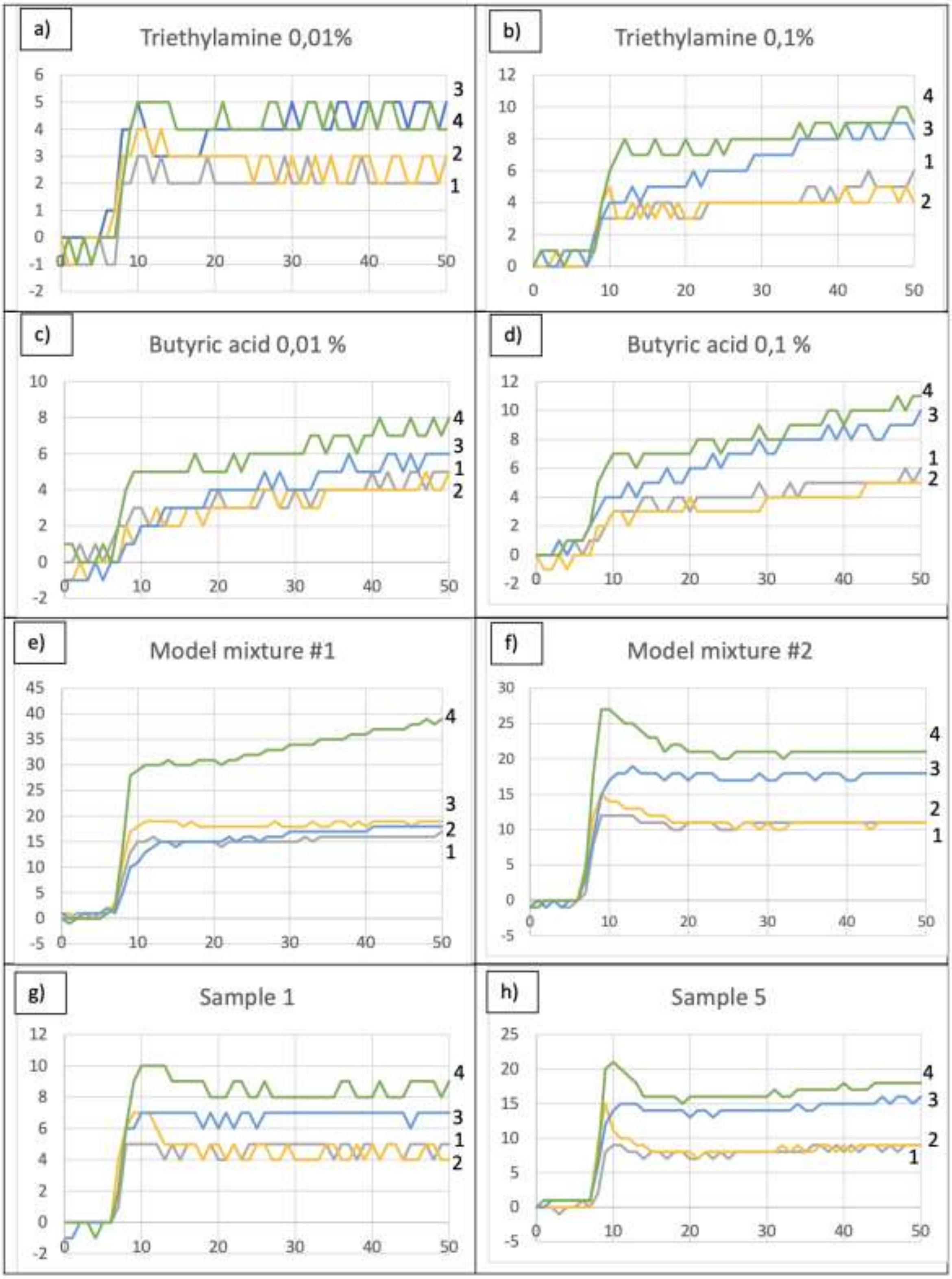

Figure S1: Chronofrequency grams of sensors with polycomposite coatings (PEG-2000/TX-100—1, TX-100/TW—2, PEGA/18-crown-6—3, 18-crown-6/PDEGS—4) when measuring the gas phase over: solution of triethylamine 0.01% (a) and 0.1% (b), butyric acid 0.01% (c) and 0.1% (d), model mixture 1 (e), model mixture 2 (f), sample 1 of exhaled breath condensate (g), sample 5 of exhaled breath condensate (h).
